# Successful pregnancy with donor eggs in-vitro fertilization after premature ovarian insufficiency in a tertiary hospital in a low-income setting: a case report

**DOI:** 10.1186/s40738-016-0028-3

**Published:** 2016-11-21

**Authors:** Thomas Obinchemti Egbe, Carine Youta Wafo, Berthe Bebey Bollo, Christian Pany, Monique Jong Onomo, Guy Sandjon

**Affiliations:** 1 Faculty of Health Sciences, University of Buea, P.O. Box 63, Buea, Cameroon; 2 Department of Obstetrics and Gynecology Douala General Hospital, P.O. Box 4856, Douala, Cameroon; 3 Clinique de l’Aeroport, Douala, Cameroon; 4 CMA Congo, Douala, Cameroon; 5 Biomedicam Laboratory, Douala, Cameroon

**Keywords:** Premature ovarian insufficiency, In-vitro fertilization, Donor eggs, Recipient, Cesarean section

## Abstract

**Background:**

Premature Ovarian Insufficiency (POI) is classically defined as 4–6 months of cessation of menses (amenorrhea) in women under 40, associated with menopausal level of serum gonadotropins FSH > 40 IU/L and hypo-estrogenism and is also referred to as hypergonadotropic hypogonadism. This disorder can manifest as primary amenorrhea without the onset of menses (menarche), or as secondary amenorrhea after menarche and pubertal development. The diagnosis of this condition in Cameroon is sometimes difficult because of the high cost of hormonal assays and the few laboratories offering these services.

**Case presentation:**

The patient was a 38-year-old G2P0020, blood group O Rh positive, genotype AA and BMI 19 kg/m2 who came to our service because of secondary amenorrhea and infertility of 2 years’ duration. She has a history of pulmonary tuberculosis that was treated in Cameroon. After laparoscopy and hormonal profile, the diagnosis of premature ovarian insufficiency was reached. The woman underwent a successful donor egg in-vitro fertilization cycle and delivered a female fetus. Two years later YE requested IVF with autologous eggs, which was not possible, and since then she has remained with one child.

**Conclusion:**

The diagnosis of premature ovarian insufficiency is difficult in Cameroon because of the high cost of laboratory investigations and difficult access to the tests. In-vitro fertilization with donor egg is a better treatment option. Unfortunately, it is not accessible to most Cameroonians because of lack of technical ability and the existence of cultural and financial barriers.

## Background

The median age of natural menopause in African-American women is 49.4 years. This is 6–12 months lower than the average age for caucasian women, 50.1 years [1, 2]. However, 1% of women under the age of 40 and 0.1% of women under 30 experience premature menopause [3]. Premature ovarian insufficiency (POI) is classically defined as 4–6 months of cessation of menses (amenorrhea) in women under 40, associated with menopausal levels of serum gonadotropins FSH > 40 IU/L and hypo-estrogenism, also referred to as hypergonadotropic hypogonadism. This disorder can manifest as primary amenorrhea, without the onset of menses (menarche) or as secondary amenorrhea after menarche and pubertal development [4, 5]. The diagnosis of this condition in Cameroon is sometimes difficult because of the high cost of hormonal assays and the few laboratories offering these services.

POI leads to infertility and hypo-estrogenism which generates problems linked with the target organs that contain estrogen receptors (osteoporosis, cardiovascular diseases, or neurodegenerative diseases). In current gynecologic practice, hypo-estrogenism can be managed with hormone replacement therapy (HRT) that is given until the age of physiological menopause. On the other hand, fertility cannot be regained when the diagnosis of POI (or end-stage POI) has been made [6, 7]. We report here a case of secondary infertility due to premature ovarian insufficiency (POI) treated by donor egg in-vitro fertilization in Douala, Cameroon.

## Case presentation

The patient was a 38-year-old G2P0020, blood group O Rh positive, genotype AA with a BMI of 19 kg/m2. She presented to our service because of secondary amenorrhea and infertility of 2 years’ duration. She had a history of pulmonary tuberculosis that was treated for 6 months in Cameroon with rifampicin, ethambutol, isonazide and pyrazinamide for 2 months and rifampicin and isonazide plus vitamin B to prevent peripheral neuropathy for a further 4 months. There was no family history of premature ovarian insufficiency or auto-immune disease. Her history was notable for laparoscopic surgery in Paris, France, in 1998, where 2 small fibroids were removed and biopsy done on both ovaries. Histology showed right ovarian dystrophy with no primordial ovarian follicles or corpus luteum scar, and uterine leiomyomata. There was left ovarian atrophy with one primordial follicle and one atretic follicle. The diagnosis of Stein-Leventhal syndrome was incorrectly made. Her hormonal profile done in France, revealed an estradiol of 15ρg/ml, FSH levels of 68.2 IU/ml and 92.03 IU/ml, LH 61.7 IU/ml and 43.91, and inhibin B 15ρg/ml (normal >45ρg/ml). Complementary laboratory test results done in Cameroon revealed Prolactin 91.57 mIU/l, T3 1.53 nmol/l, T4 90.68 nmol/ml, THS (ultra sensitive) 0.65 ng/ml and Testosterone 0.06 ng/ml.

The patient underwent the progesterone challenge test with Synergon® (progesterone, estrone) which was negative. The husband had normal semen parameters. The diagnosis of premature ovarian insufficiency was discussed and the couple was counseled to undergo egg donor In-vitro fertilization.

After obtaining informed consent, we matched the anonymous donor-controlled ovarian stimulation cycle with the recipient’s endometrial preparation. For this purpose, we used the short agonist protocol (triptorelin (Decapeptyl®) 0.1 mg/day plus menotropin (Menogon®) 150 IU/day) starting from the third day of the donor’s cycle untill we obtained three leading follicules of 17–18 mm in diameter. The donor was monitored with standard trans-vaginal sonography and serum estradiol levels.

We prepared the patient’s (recipient YE) endometrium for embryo transfer with estradiol valerate (progynova®) 8 mg/day in three divided doses for 14 days starting from the first day of the donors menses. A trans-vaginal sonogram was performed every 3 days to monitor the endometrial thickness until we obtained an endometrial thickness of about 14 mm. The husband’s sperm was prepared with the density gradient centrifugation as previously described [8–10]. A total of four fresh donor oocytes were obtained and were inseminated and the patient (recipient) began progesterone (utrogestan® vaginal tablets) 600 mg/day from the day of oocyte insemination. Two fresh 8-cell (grade A) embryos were transferred to the recipient YE (patient) on day 3 of insemination after the standard IVF procedure. The remaining two (grade C) embryos were discarded for lack of facilities for gamete freezing at the time. (However, since 2012 our IVF centre has the facilities to freeze gametes and embryos.)

**Fig. 1:**
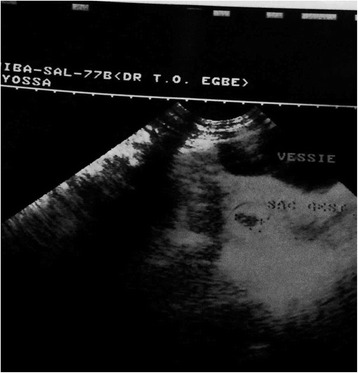
Gestatational Sac of Patient at 6 weeks gestation

**Fig. 2:**
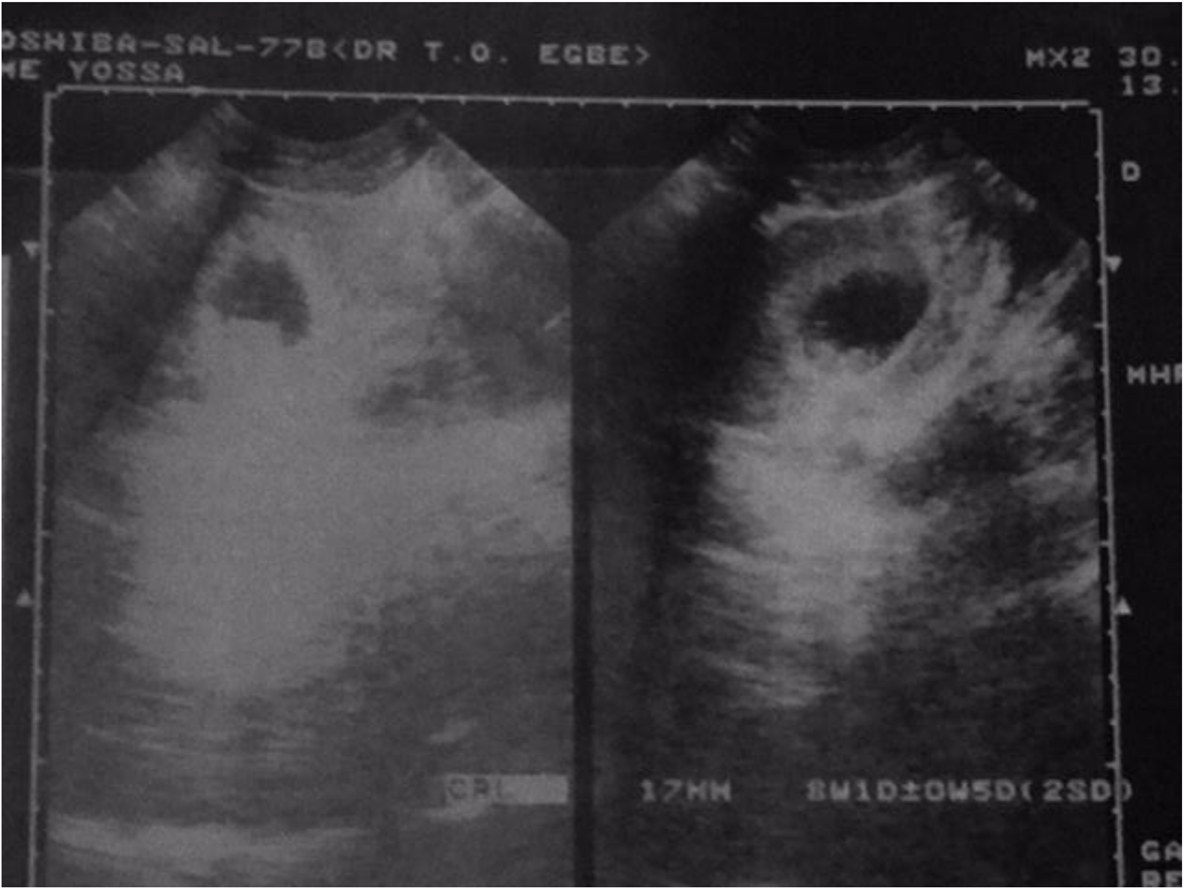
Gestational Sac of Patient at 8 weeks 1 day gestation

After embryo transfer, the recipient continued with progesterone suppositories(utrogestan®) and estradiol valerate (progynova®) and underwent her first plasma beta hCG assay 2 weeks after embryo transfer. The test result was positive, with a value of 248 IU/L. She underwent a trans-vaginal ultrasound scan 2 weeks after the positive pregnancy test that revealed an intra-uterine gestational sac of 13 mm diameter (Fig. 1). A repeat trans-vaginal ultrasound scan done about 2 weeks from the latter showed a singleton intrauterine pregnancy with a crump-rump length of 17 mm corresponding to 8 weeks 1 day gestation Fig. 2).

Her pregnancy follow-up was uneventful until *she developed preterm labour at 36 weeks 4 days gestation and fetal distress was diagnosed on cardiotocography*. She gave birth by cesarean section to a female baby who weighed 2360 g. At cesarean section, the presentation was breech with two turns of nuchal cord. One and 5-min Apgar scores were 8 and 10, respectively. The baby was sent to the neonatal intensive care unit (NICU) for follow up. Two years later, YE requested for IVF with autologous eggs which, it was explained, was not possible. She is now living with one child.

## Discussion

Premature ovarian insufficiency is a difficult diagnosis to make in current gynecologic practice in Cameroon. In majority of cases the underlying cause is not identified. The diagnosis of POI on this patient was based on secondary amenorrhea of 2 years’ duration and elevated FSH (61.7 IU/ml and 92.03 IU/ml) on two occasions 8 weeks apart and low estrogen (<15ρg/ml) in a 38-year-old lady. This unfortunate patient underwent laparoscopy with ovarian biopsies, which in fact did confirm the absence of follicles, but was not diagnosed correctly as consistent with ovarian insufficiency. Her findings are consistent with the literature [4, 5]. In this case, the patient had normal secondary sexual organ development (regular menstrual patterns, hair distribution etc.) and no physical signs of hyper-androgenism. There was no family history of POI before the condition. She developed secondary amenorrhea only after she was diagnosed and treated for pulmonary tuberculosis.

Genital tuberculosis has been associated with menstrual disorders [11] and premature ovarian insufficiency [12]. A prior study evaluated the menstrual patterns of 120 women with genital tuberculosis diagnosed by histopathology. The common menstrual disorder was oligo-hypomenorrhea found in 54.0% cases, menorrhagia in 19.0% cases, and post menopausal bleeding in 1.6% cases. A history of amenorrhea was present in 14.3% cases. There were 8 cases of secondary amenorrhea and one case of primary amenorrhea [11]. A direct cause–effect relationship between tuberculosis and premature ovarian insufficiency has not yet been established [13]. Most women with premature ovarian insufficiency report seeing three or more different clinicians before diagnosis. Furthermore, women with spontaneous premature ovarian insufficiency perceived a need for more aggressive evaluation of secondary amenorrhea and oligomenorrhea [12].

The major concern of this couple was infertility, especially given the age and parental status of the patient (38 years old, with no living child). She may have been counseled to wait for some years to obtain a spontaneous pregnancy as has been reported in other studies [14, 15]. However, IVF with donor egg was immediately proposed to this patient because of her advanced age and because it is the treatment of choice for POI [16–18].

The Lagos University Teaching Hospital team of Giwa-Osagie at al. were the first in Africa to produce documented pregnancies through IVF in 1984 and 1986, and a live birth in 1989. The successes from IVF at the Lagos University team were the first in West, East and Central Africa. Now all ART units performing IVF in West, Central and East Africa are in the private sector, or are incorporated as foundations [19]. However, egg-donor IVF has been documented only in Nigeria [19]. In Cameroon, there are two IVF centres in the private sector in Douala [20], and a third centre that has just been opened in the public sector in Yaounde. Despite these centres, there has been no documented evidence of donor egg IVF services in Cameroon in the existing medical literature [19].

The cost of IVF in most sub-Saharan African countries ranges between 2500 and 3500 USD including drugs [21]. This cost is prohibitive in a country where the monthly income ranges between 21.83 and 94.54 USD [22, 23], and where there is no insurance coverage for IVF as compared to countries like Israel, France and Belgium that have elaborate coverage [20]. Such rates constitute a hurdle for infertile couples wishing to undergo IVF treatment. There is need for a cost-effective, simplified, assisted reproduction treatment protocol for low-income countries like Cameroon [24].

There is no formal egg donation program in Cameroon. We usually pair a donor (woman ≤30 years serologically tested and programmed for IVF) with a recipient. The recipient pays for the IVF charges of the donor with an additional fee of 423 USD irrespective of the number of oocytes donated). The donor only pays for the drugs used for ovarian hyperstimulation. This helps in reducing the cost of the procedure for both the donor and the recipient. This amount is small compared with that reported by some centres in the USA [25].

In Cameroon, there is no legislation on the practice of IVF. The IVF centre in Douala, Cameroon applies the guidelines as practiced in France because we work in collaboration with Dr Guy Casutto’s Drouot Laboratory in Paris, France. Attitudes of users of IVF vary depending on cultural and especially religious backgrounds. Cameroon has a variety of denominations that range from Christianity through Islam to Animism. In 1987, the Roman Catholic Church issued a document “Donum vitae” which stated that IVF was illicit and was not to be used by the faithful [20]. Roman Catholic Christians typically abide by that principle. Other religious groups have different attitudes.

Another option for treatment of POI, is ovarian tissue transplantation, which has been used between monozygotic twins discordant for premature ovarian insufficiency. After unsuccessful egg-donation therapy, the sterile twin received a transplant of ovarian cortical tissue from her sister by means of a mini-laparotomy. Within three months of transplantation, the recipient’s cycles resumed and serum gonadotropin levels fell to the normal range. During the second cycle, she conceived and her pregnancy progressed uneventfully [26]. This was not an option for the index patient as she did not have a twin. Ovarian tissue transplantation has never been attempted in Cameroon. This could, however, be a subject for further research.

IVF centres in sub-Saharan Africa (SSA) now practice most of the ART procedures: artificial insemination with husband’s sperm (AIH), artificial insemination with donor sperm (AID), IVF, intra-cytoplasmic sperm injection (ICSI), Donor gamete IVF and freezing (cryopreservation) of gametes like most centres in Europe and USA [19].

The major difference between IVF centres in SSA and those in Europe and the USA is that centres in Europe and the USA have the capacity to perform pre-implantation genetic diagnosis [27, 28] and a larger number of IVF cycles per annum. In a study of 21 countries in the ART register in Europe, a total of 399 020 ART cycles were performed in a population of 373.8 million, corresponding to 1067 cycles per million inhabitants [29]. The take-home baby rate per cycle is 26.6% in Europe and 40.7% in the USA [30], and are greater than in SSA centres (50–159 cycles per annum, take-home babies 10–15.8%) [19].

There is not only high cost of ART as compared to the average income of Cameroonians, there is also lack of knowledge about the different ART procedures (IUI and IVF) and inaccessibility to IVF centres. All three IVF centres in Cameroon are located in the two main cities, Yaounde and Douala. It is difficult for the rural dwellers to have access to these centres. Traditional beliefs and customs may also play a role. Generally, what is not known is accepted with difficulty. All these factors make donor egg IVF a rare procedure in Cameroon. Furthermore, the country lacks qualified reproductive endocrinologists and embryologists, mainly because there is no sustained training program for them. Most of the reproductive endocrinologists in Cameroon have been trained either in Europe or in the USA.

Finally, lawmakers and educators usually support research and investment in other aspects of reproductive health including contraception, HIV/AIDS prevention and maternal mortality reduction. It is generally thought that the problem of overpopulation can only be solved by good family planning programs and education. Therefore, some believe that the infertile couple should be encouraged to courageously accept their condition of childlessness rather than be offered intervention [31]. There is therefore little or no emphasis as regards infertility and assisted reproduction in Cameroon.

The index patient came to our infertility clinic 2 years later to seek for IVF with her own eggs because she never resumed menstruation. It has been reported that although an unknown donor was felt to be a much safer option, as compared to adoption, in terms of protecting the mother–child relationship, the lack of information raised concerns about what genetic condition might emerge in the child. There is wide variation in how much women are preoccupied by the donor screening and matching process [32]. However, we did not have this problem with the index case.

Most of the ethnic cultures in Cameroon and in most of SSA do not accept childlessness [19, 31]. To that effect, traditionally, couples seek for children from relatives (a sister bearing a child with the husband of another sister who could not conceive or a brother impregnating the wife of an infertile brother, etc.). All these practices are kept as family secrets, the reason being to maintain the genetic constitution of the family and avoid hereditary ailments like madness, drunkenness, etc. Cameroonian society has easily adopted ART procedures. Nevertheless, the offspring of this technology, including relatives and friends, are usually blinded to the facts of their conception [19, 31]. Furthermore, concerning adoption, it has been reported that in Cameroon the level of knowledge is high among the educated class but many women lack knowledge regarding how and where to go in order to effect an adoption. The attitude towards adoption is favourable but the practice of adoption is still limited [33].

### Limitations

Some patients in Cameroon may not accept donor egg IVF for cultural or religious reasons. This was not the case with our patient. The cost of IVF (about 3279 USD) per IVF cycle in Cameroon might be a limiting factor for most couples, but our couple was of high socio-economic standing and could readily afford the procedure. For economic reasons, many patients are not able to afford IVF in Cameroon. Hormonal and immunologic diagnosis, including genetic studies of patients, is a further limitation. Quite often, the tests cannot be done in Cameroon, which means that the samples have to be sent to Europe or to the USA for analysis. In the case of our patient, some of the follow-up and diagnosis was done in France. This led to higher cost and a longer time to obtain the results and initiate treatment.

The strength of the case is that it conforms with the current literature in that we transferred two fresh 8-cell day 3 embryos to obtain a singleton pregnancy in one cycle. Current practice recommends ideally single embryo transfer (SET) in two consecutive cycles, although two embryos transferred in one cycle affords the same pregnancy rate but higher twinning rate [34–37]. Assisted reproductive technology (ART) is not popular in Cameroon and in much of sub-Saharan Africa, the main reason being the lack of equipment and trained staff, and financial and cultural barriers. There is some bias as some believe that a child obtained from ART is not a normal child.

## Conclusion

Premature ovarian insufficiency remains a dilenma in Cameroon. The gold standard should be good counselling in favour of donor-egg IVF for those who require children and can afford it, and HRT to avoid complications of hypoestrogenism in the long term. ART is not accessible to most Cameroonians because of lack of technical ability, and cultural and financial barriers.

## Abbreviations

ART: Assisted reproductive technology
HRT: Hormone replacement therapy
ICSI: Intracytoplasmic sperm injection
IUI: Intrauterine insemination
IVF: In-vitro fertilization
POI: Premature ovarian insufficiency
SET: Single embryo transfer
SSA: Sub-saharan Africa
USA: United States of America
USD: United States Dollars.

## References

[1] Gold EB, Bromberger J, Crawford S, Samuels S, Greendale GA, Harlow SD, Skurnick J (2001). Factors associated with age at natural menopause in a multiethnic sample of midlife women. Am J Epidemiol.

[2] Bromberger JT, Matthews KA, Kuller LH, Wing RR, Meilahn EN, Plantinga P (1997). Prospective study of the determinants of age at menopause. Am J Epidemiol.

[3] Coulam CB, Adamson SC, Annegers JF. Incidence of premature ovarian failure. Obstet Gynecol. 1986;67(4):604-6. http://journals.lww.com/greenjournal/pages/default.aspx. 3960433

[4] Goswami D, Conway GS (2005). Premature ovarian failure. Hum Reprod Update.

[5] Kalantaridou SN, Davis SR, Nelson LM (1998). Premature ovarian failure. Endocrinol Metab Clin North Am.

[6] Welt CK (2008). Primary ovarian insufficiency: a more accurate term for premature ovarian failure. Clin Endocrinol (Oxf).

[7] Gleicher N, Weghofer A, Oktay K, Barad D (2009). Do etiologies of premature ovarian aging (POA) mimic those of premature ovarian failure (POF)?. Hum Reprod.

[8] Michelmann HW. Semen Preparation Techniques. In Gautam Nandkishore Allahbadia, editor. Intrauterine Insemination. London: Taylor & Francis Group; 2005. p. 197-217.

[9] Organization WH (2010). WHO laboratory manual for the examination and processing of human semen.

[10] Henkel RR, Schill W-B (2003). Sperm preparation for ART. Reprod Biol Endocrinol RBE.

[11] Samal S, Gupta U, Agarwal P (2000). Menstrual disorders in genital tuberculosis. J Indian Med Assoc.

[12] Alzubaidi NH, Chapin HL, Vanderhoof VH, Calis KA, Nelson LM (2002). Meeting the needs of young women with secondary amenorrhea and spontaneous premature ovarian failure. Obstet Gynecol.

[13] Shapira Y, Agmon-Levin N, Shoenfeld Y (2009). Mycobacterium Tuberculosis, Autoimmunity, and Vitamin D. Clin Rev Allergy Immunol.

[14] Anna Liza R, Alik RZ, Ahmad Murad Z, Ghazali I (2008). Spontaneous twin pregnancy in premature ovarian failure. Med J Malaysia.

[15] Fujii S, Ikeda S, Tachizaki T, Kagiya A, Saito Y (1993). Successful pregnancy in a woman with premature ovarian failure. Asia-Ocean J Obstet Gynaecol AOFOG.

[16] Lydic ML, Liu JH, Rebar RW, Thomas MA, Cedars MI (1996). Success of donor oocyte in in vitro fertilization-embryo transfer in recipients with and without premature ovarian failure. Fertil Steril.

[17] Check JH, O’Shaughnessy A, Lurie D, Fisher C, Adelson HG (1995). Evaluation of the mechanism for higher pregnancy rates in donor oocyte recipients by comparison of fresh with frozen embryo transfer pregnancy rates in a shared oocyte programme. Hum Reprod Oxf Engl.

[18] Oyesanya OA, Olufowobi O, Ross W, Sharif K, Afnan M (2009). Prognosis of oocyte donation cycles: a prospective comparison of the in vitro fertilization-embryo transfer cycles of recipients who used shared oocytes versus those who used altruistic donors. Fertil Steril.

[19] Giwa-Osagie OR. ART in developing countries with particular reference to sub-Saharan Africa. In Vayena E, Rowe PJ, Griffin PD, editors. Current Practices and Controversies in Assisted Reproduction. Reprot of a meeting on Medical, Ethical and Social Aspects of Assisted Reproduction held at the WHO Headquarters in Geneva Switzerland 17-21 September 2001. Geneva: World Health Organisation; 2002. p. 22-27.

[20] Ory SJ, Devroey P, Banker M, Brinsden P, Buster J, Fiadjoe M, Horton M, Nygren K, Pai H, Le Roux P, Sullivan E (2014). International Federation of Fertility Societies Surveillance 2013: preface and conclusions. Fertil Steril.

[21] Pilcher H (2006). IVF in Africa: Fertility on a shoestring. Nature.

[22] Cameroon map of Map of Cameroon. Average Monthly Rural Income by Region [2010 Data] by Province - TargetMap [http://www.targetmap.com/viewer.aspx?reportId=15252].

[23] Tambi NE (2001). Analysis of household attitudes toward the purchase of livestock products and fish in Cameroon. Agric Econ.

[24] Ombelet W, Campo R (2007). Affordable IVF for developing countries. Reprod Biomed Online.

[25] Egg Donor Program for Donor : CHR [https://www.centerforhumanreprod.com/egg-donation/program/].

[26] Silber SJ, Lenahan KM, Levine DJ, Pineda JA, Gorman KS, Friez MJ, Crawford EC, Gosden RG (2005). Ovarian Transplantation between Monozygotic Twins Discordant for Premature Ovarian Failure. N Engl J Med.

[27] Baruch S, Kaufman D, Hudson KL (2008). Genetic testing of embryos: practices and perspectives of US in vitro fertilization clinics. Fertil Steril.

[28] Thornhill AR, Geraedts JP, Harper JC, Harton GL, Lavery SA, Moutou C, Robinson MD, Schmutzler AG, Scriven PN, Sermon KD (2005). ESHRE PGD Consortium “Best practice guidelines for clinical preimplantation genetic diagnosis (PGD) and preimplantation genetic screening (PGS).”. Hum Reprod.

[29] Ferraretti Ap, Goossens V, Kupka M, Bhattacharya S, de Mouzon J, Erb K, Korsak V, Andersen NA. Assisted reproductive technology in Europe, 2009: results generated from European registers by ESHRE. Human Reproduction; 2013. p. 1-14. doi:10.1093/humrep/det278. 10.1093/humrep/des023 PMC3303494 22343707

[30] IVF Success Rates Plateau in Europe but Not in the US: CHR. Fertility Updates. 2014. https://www.centerforhumanreprod.com/services/.

[31] Tangwa GB. ART and African sociocultural practices: worldview, belief and value systems with particular reference to francophone Africa. Curr Pract Controv Assist Reprod Geneva Switz World Health Organ. 2002;55–59.

[32] Stuart-Smith SJ, Smith JA, Scott EJ (2012). To know or not to know? Dilemmas for women receiving unknown oocyte donation. Hum Reprod.

[33] Nguefack CT, Ourtching C, Gregory HE, Priso EB (2014). Knowledge, Attitudes and Practices of Infertile Women on Child Adoption in Douala (Cameroon). Open J Obstet Gynecol.

[34] Ashrafi M, Madani T, Movahedi M, Arabipoor A, Karimian L, Mirzaagha E, Chehrazi M (2015). Increasing The Number of Embryos Transferred from Two to Three, Does not Increase Pregnancy Rates in Good Prognosis Patients. Int J Fertil Steril.

[35] Medicine TPC of the S for ART and the PC of the AS for R (2006). Guidelines on number of embryos transferred. Fertil Steril.

[36] Pandian Z, Marjoribanks J, Ozturk O, Serour G, Bhattacharya S (2013). Number of embryos for transfer following in vitro fertilisation or intra-cytoplasmic sperm injection. Cochrane Database Syst Rev.

[37] Fechner AJ, Brown KR, Onwubalili N, Jindal SK, Weiss G, Goldsmith LT, McGovern PG (2014). Effect of single embryo transfer on the risk of preterm birth associated with in vitro fertilization. J Assist Reprod Genet.

